# IoT based real-time water quality monitoring system in water treatment plants (WTPs)

**DOI:** 10.1016/j.heliyon.2024.e40746

**Published:** 2024-11-29

**Authors:** H.M. Forhad, Md. Ripaj Uddin, R.S. Chakrovorty, A.M. Ruhul, H.M. Faruk, Sarker Kamruzzaman, Nahid Sharmin, AHM Shofiul Islam Molla Jamal, Md. Mezba-Ul Haque, AKM M Morshed

**Affiliations:** a Pilot Plant and Process Development Centre (PP&PDC), Bangladesh Council of Scientific & Industrial Research (BCSIR), Dhaka, 1205, Bangladesh; b Institute of National Analytical Research and Service (INARS), Bangladesh Council of Scientific and Industrial Research (BCSIR), Dhanmondi, Dhaka, 1205, Bangladesh; c Pagla Sewage Treatment Plant Division, Dhaka, WASA, Bangladesh; d Department of Mechanical Engineering, Bangladesh University of Engineering and Technology (BUET), Dhaka, Bangladesh

**Keywords:** Internet of things (IoT), Programmable logic controller (PLC), Water quality, Real-time monitoring

## Abstract

This study presents the development and implementation of an Internet of Things (IoT)-based real-time water quality monitoring system tailored for water treatment plants (WTPs). The system integrates advanced sensor technologies to continuously monitor key water quality parameters such as pH, dissolved oxygen (DO), total dissolved solids (TDS), and temperature. Data collected by these sensors is transmitted through a robust communication network to a centralized monitoring platform that utilizes cloud-based storage and analytics. The system's design includes a PLC-based control mechanism, allowing for flexible setup modifications and the easy addition of new monitoring parameters. The IoT-based system, powered by a low-energy 29-W configuration, offers accurate and reliable data with a minimal error margin of 0.1–0.2 across various parameters. The research highlights the system's ability to provide real-time alerts, historical data logging, and remote monitoring, all of which contribute to enhanced operational efficiency, proactive maintenance, and informed decision-making. This innovative approach to water quality management not only improves the effectiveness of WTP operations but also ensures environmental sustainability and public health safety. The study underscores the significant potential of IoT technologies in revolutionizing water quality monitoring practices.

## Highlights

• The measurement accuracy of TDS, water temperature, and DO is 0.1, while pH is 0.2.
• Data is sent via a robust network to a cloud-based system for storage and access.
• Easily determine the water quality hourly, year-round on-site and remotely.
• PLC-based, robust controller for monitoring, control, optimization, allows easy setup modification and adding sensors for new parameters.
• The system consumes just 29 W and costs only $2445.

## Graphical abstract

**Figure:**
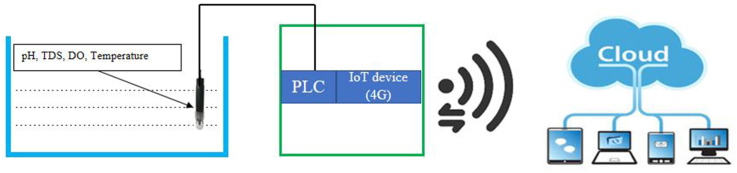

## 1 Introduction

Water treatment plants are critical infrastructure components tasked with ensuring the supply of safe and clean drinking water to communities [1]. These facilities employ various processes such as filtration, disinfection, and chemical treatment to remove contaminants and pathogens from raw water sources [2]. However, maintaining consistent water quality standards throughout the treatment process is a complex challenge that requires continuous monitoring and management (WHO, 2011) [3]. Traditional methods of water quality monitoring in WTPs typically involve manual sampling at predetermined intervals, followed by laboratory analysis of collected samples [4]. While these methods provide valuable insights into water quality parameters, they are limited by their episodic nature and the time required for analysis. This inherent delay in obtaining data can impede timely responses to emerging water quality issues, potentially jeopardizing public health and safety [5]. The emergence of IoT technology has revolutionized the field of water quality monitoring by enabling real-time, remote monitoring capabilities [6]. IoT-based systems leverage a network of sensors and connected devices deployed throughout the water treatment process to continuously measure key parameters such as pH, turbidity, DO, conductivity, temperature, and TDS levels [7]. These sensors transmit data wirelessly to a centralized monitoring platform, where it can be analyzed in real-time to detect deviations from established quality standards. Wireless communication protocols such as Wi-Fi, LoRaWAN, and cellular networks are commonly employed to facilitate data transmission [8,9]. The researcher explored the use of LoRaWAN technology for long-range, low-power data transmission in IoT-based water quality monitoring systems, demonstrating its suitability for WTP applications.

The vast amount of data generated by IoT sensors necessitates the use of advanced analytics techniques to extract actionable insights for water quality management [10]. Data analytics methods such as anomaly detection, trend analysis, and predictive modeling enable operators to identify deviations from normal operating conditions, predict potential issues, and optimize treatment processes [11]. The Researchers [12,13] showcased the efficacy of machine learning algorithms in analyzing water quality data and predicting water quality parameters in real-time, thereby enhancing decision support capabilities in WTPs. Despite the potential benefits of IoT-based water quality monitoring systems, several challenges and considerations must be addressed during implementation [14]. These include power supply constraints for remote sensor nodes, connectivity issues in remote or underground locations, data security and privacy concerns, and regulatory compliance with water quality standards [15]. Strategies for overcoming these challenges, such as the use of renewable energy sources, robust encryption protocols, and adherence to industry standards, have been explored in studies [16,17]. The implementation of an IoT-based real-time water quality monitoring system brings several benefits to WTPs [18]. Firstly, it enables early detection of anomalies or contamination events, allowing for prompt response measures to mitigate risks to public health. Secondly, the continuous monitoring capability improves operational efficiency by optimizing chemical dosing, reducing energy consumption, and minimizing wastage. Moreover, the availability of comprehensive data sets facilitates trend analysis, predictive maintenance, and informed decision-making for long-term infrastructure planning [19].

Water is the most important element of living organisms [20]. Water pollution control is a major concern in Bangladesh because huge industrial development is going on in Bangladesh [21,22]. River water quality is deteriorating day by day. All the waste water finally mixed with the river water [23]. To control water pollution, monitoring of all running ETPs are running effectively is a great challenge for DoE [24]. To overcome this problem, IoT-based ETP monitoring can play a vital role. Despite the growing body of literature on IoT-based real-time water quality monitoring systems, there exists a notable research gap concerning the integration of advanced sensor technologies specifically tailored for comprehensive water quality assessment in WTPs. Researchers [[25], [26], [27]] provide a comprehensive review of IoT-based water quality monitoring systems. While the review covers various aspects of sensor technologies and data analytics methods, it lacks specific focus on the challenges and requirements unique to WTP environments. Consequently, there is a research gap in the literature regarding the optimization and integration of advanced sensor technologies explicitly designed for use within WTPs to ensure comprehensive and accurate water quality assessment throughout the treatment process. Addressing this research gap is crucial for advancing the development and deployment of IoT-based real-time water quality monitoring systems in WTPs.

This is a unique approach that is Programmable Logic Controller (PLC) based, most of the researchers done this thing through microcontrollers. PLC is a robust controller for monitoring, control and optimizing a process. By using PLC, it is very easy to modify the setup. In this existing setup it is possible to add more monitoring parameters by adding the respective sensor. For that, only the PLC programming need to be modified. In the hardware part, only the respective sensor need to be added with the existing setup. Real-time trends and historical trends also novel approach. In most of the researchers has not developed such kind of facilities. This setup can also be utilized to monitor the various points river for the river water quality. By utilizing this setup a central monitoring system can possible to developed through which multiple ETPs as well as various points of river can possible to monitor through a cloud server. The integration of IoT technology into water treatment plants offers significant advantages in terms of enhanced monitoring capabilities, improved responsiveness, and informed decision-making. This paper explores the design, implementation, and benefits of an IoT-based real-time water quality monitoring system in WTPs, highlighting their potential to revolutionize water quality management practices and safeguard public health.

## 2 Methods and materials

### 2.1 IoT device for monitoring the water quality

**Fig. 1:**
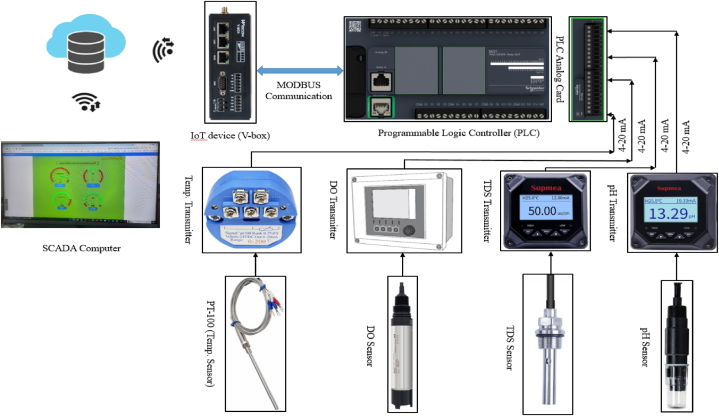
Block diagram of the system.

To develop the IoT based real-time water quality monitoring system, it has divided into three parts:

hardware, IoT-based configuration, and practical implementation. In the hardware section, water quality monitoring sensors and controllers have been collected to accomplish the project as specified. These include pH (model: SUP-PH6.0), TDS (model: SUP-TDS210-B), DO (model: COS61D-AAA1A3 and CM442-3PQ7/0), and temperature sensors (model: PT100). Additionally, an IoT device, a PLC with model TM221CE24R, a PLC Analog card (model: TM3AI8), an AC to DC converter (model: NDR-120-24), and other necessary equipment are included. In Fig. 1 over all block diagram has shown the system for monitoring the water quality parameters.

### 2.2 Circuit diagram of IoT water quality monitoring system

**Fig. 2:**
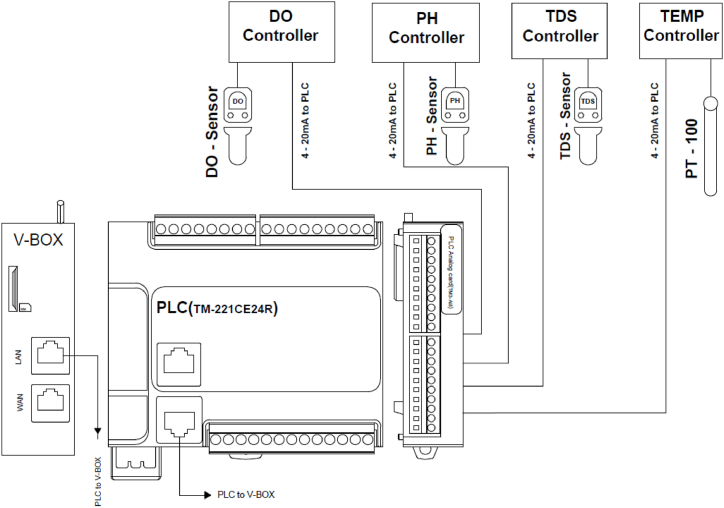
Circuit diagram of IoT based water quality monitoring system.

All the sensor has checked with the standard sample and the error was found within the range of 1–2%. Each of the sensors has its own controller which gives an output of 4–20 mA. These 4–20 mA signals has transferred to an analog card of the PLC. In the PLC analog card four channels has configured of 4–20 mA out of eight (refer to Fig. 2 and Table 2). Then a PLC program has developed to monitor these four parameters. Also, a start/stop switch has been added to the program to run or stop the system remotely. Then an IoT device has used to send the data from PLC to the cloud server where the IoT device has a MODBUS TCP/IP communication with the PLC. The used IoT device from Wecon Technology is named as V-box. Modbus is a widely used communication protocol in industrial automation systems. It allows various devices, such as sensors, actuators, and controllers, to communicate with each other over different types of networks. Modbus TCP/IP is suitable for communication over Ethernet networks. Some other industrial communication protocols are mentioned on Table 1 which can also be useable for this system.

**Table 1:** Comparison of among the various industrial communication protocol systems.

Communication protocol Name	Type	Features (advantages and disadvantages)
**Modbus**	Serial and Ethernet-based.	Simplicity, wide adoption, flexibility. Limited speed, no built-in security, no standardized device profile.
	Modbus RTU: Operates over RS-232 or RS-485.	
	Modbus TCP/IP: Operates over Ethernet networks.	
**Profibus (Process Field Bus)**	Serial. Profibus DP: Designed for fast communication between controllers and field devices.	High speed, scalability, robustness. Complexity, wiring costs, limited expansion.
	Profibus PA: Used in process automation, supporting intrinsic safety in hazardous environments.	
**Profinet**	Ethernet-based.	High performance, integration, scalability. Complexity, cost, learning curve.
**EtherNet/IP (Ethernet Industrial Protocol)**	Ethernet-based.	Interoperability, high bandwidth, real-time capabilities. Network traffic, security concerns, complex setup.
**CAN (Controller Area Network)**	Serial.	Reliability, real-time communication, low cost. Limited speed, limited distance, limited number of devices.
**HART (Highway Addressable Remote Transducer)**	Hybrid (Analog and Digital).

**Table 2:** Electrical specifications of sensors and equipments.

	pH	DO	TDS	Temperature	PLC	PLC Analog card	AC to DC Converter	IoT device
Supply Voltage	220 VAC ±10 %	100–230V	220 VAC ±10 %	24 V	100-240 VAC	100-240 VAC	100-240 VAC	24VDC
Range	0–14	0–20 mg/L	0–1000 ppm	0-200 °C	14 DI, 10 DO, 2 AI	8 AI	2.6 A	2 LAN, 1 WAN Port/Ethernet, Wi-Fi, 4G Connectivity
Output	4–20 mA	4–20 mA	4–20 mA	4–20 mA		+-10 V, 0–10 V, 0–20 mA, 4–20 mA	24 V	

A 4g enable subscriber identity/identification module (sim) card has inserted and configured to the IoT device for sending the data from PLC to cloud server through the IoT device. The 4g enable sim card and network has used from Airtel company.

### 2.3 Real-time water quality monitoring approach

In IoT based configuration part, first the IoT (V-box) device has bound through the V-net software.

**Fig. 3:**
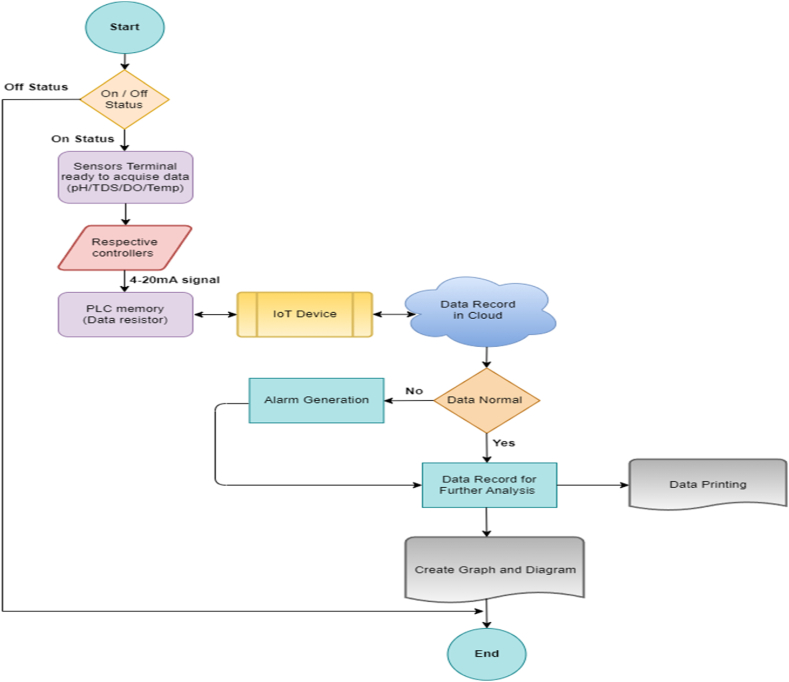
Over all Flowchart the process.

**Fig. 4:**
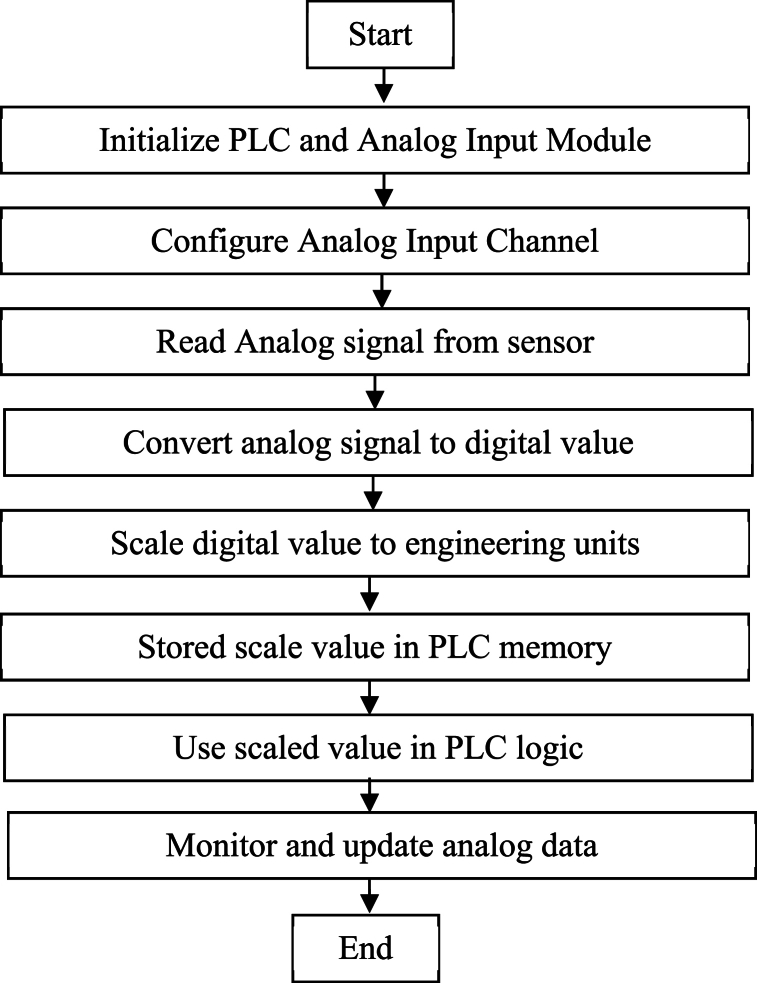
Flowchart for taking analog data to PLC.

V-net is an internet-based configuration platform for the IoT device (V-box). To access the V-net first registration has done by setting a unique ID and password for login to the V-net platform. Then configuration has done for real-time data, alarm, and historical data. After completing the configuration for real-time data, alarm and historical data web cloud configuration has done for monitoring and control from the cloud server. Web cloud configuration is a graphical representation for monitoring real time data, historical data acquisition and trend display systems. The process flowchart has shown in Fig. 3 and PLC flowchart shown in Fig. 4.

Illustrates the previous works especially focusing on the hardware and software used for the experiment are discussed in Table 3.

**Table 3:** Illustrates the previous IoT based WTPs systems hardware and software function.

Description	Used controller and IoT device	Software program	Limitations	Ref.
A hybrid machine learning and embedded IoT-based water quality monitoring system.	Arduino microcontroller, ESP8266 Wi-Fi module	Message Queuing Telemetry Transport (MQTT), Proteus 8 professional	Microcontroller based system is not suitable for industrial applications, limited industrial reliability, not designed for harsh environments, limited I/O and expandability	[28]
Development of LoRaWAN-based IoT system for water quality monitoring in rural areas.	Arduino microcontroller, LoRaWAN gateway	Fritzing software, Arduino IDE software		[29]
Real-time IoT-powered AI system for monitoring and forecasting of air pollution in industrial environment.	Microcontroller (Arduino MEGA), Narrowband IoT GSM	Machine learning		[30]
Advances in real time smart monitoring of environmental parameters using IoT and sensors.	Aruduino Uno-ATMega 1280, ESP8266 Wi-Fi module, GSM module	C		[[31], [32]]

### 2.4 IoT performance metrics

All the configurations which has done through v-net platform has shown on Table 4. Historical data were recorded in every minutes, According to real value trend will plot for each parameters and history trend will display according to time duration.

**Table 4:** Configuration for real time data, alarm and accuracy.

Parameter	Range	Accuracy	Working Temp	Alarm
pH (SUP-PH6.0)	0–14	±0.2	0–80 °C	Less than 5 and greater than 9
TDS (SUP-TDS210-B)	0–1000 ppm	±0.1	0–100 °C	Less than 200 ppm and greater than 900 ppm
DO (COS61D-AAA1A3)	0–20 mg/L	±0.1	0–80 °C	Less than 5 mg/L
Temperature (PT-100)	0-200 °C)	±0.1	−50–120 °C	Less than 15^0^ C and greater than 45^0^ C

The communication configuration performance between the PLC and the IoT device's broadcast status is OFF. The network type for the MODBUS PLC communication system is 'tcp client 2n′, and the IP address is 192.168.1.201. The network configuration used a local area network (LAN), internet protocol (IP) of 192.168.1.210, which enabled 4G, included a static dynamic host configuration protocol (DPCH) IP, and provided new default access point name (APN) facilities. The device has 502 ports and device station No 1. Receive timeout and retry timeout is 50 ms whereas wait timeout is 1500 ms and retry count is 2. Real-time data configuration, alarm configuration and historical data configurations were implemented using an ethernet port. The 'OFF/ON’ status and 'Start/Stop’ values were found at reading addresses 1.03 and 1.00, respectively. The real values, alarm configurations and historical data configurations for Temperature, TDS, DO, and pH were found at reading addresses 1:4 45, 1:4 35, 1:4 25, and 1:4 8, respectively. Reading address and identity (ID) auto generated by the systems and 32-bit floating systems used for historical data configurations. For graphical representation which is called cloud supervisory control and data acquisition (SCADA) for monitoring the real time data, data acquisition and trend total five screen has configured has shown in Supplementary Table S1. Cloud SCADA refers to a modern approach to SCADA systems where the core functionalities of monitoring, controlling, and data acquisition are hosted on cloud-based platforms rather than on traditional on-premises servers.

In the practical implementation part an electrical enclosure which is also called control panel has designed and manufactured. All the controller of the sensor, PLC, IoT device and all other necessary equipments has arranged in the control panel. A stand has design to hold the sensors that the sensors can takes reading at a 45^0^ angle according to manufacturer recommendation. The control panel has designed for outdoor environment. The panel has arranged in such a way that it works like plug and play. The sensor stand with the sensor need to immerse in water monitoring point and a 220 VAC supply is needed to power up the control panel.

### 2.5 Real-time water quality parameters monitoring at WTPs in Dhaka water supply and sewerage authority (WASA)

**Fig. 5:**
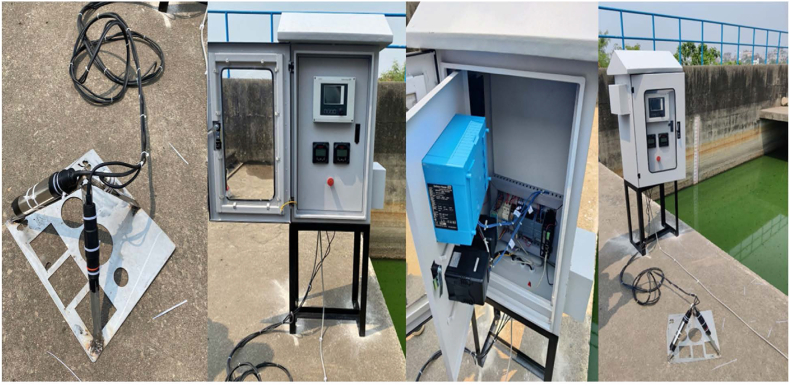
Setup of the sensor stand and control panel, (a) Sensor stand, (b) Control panel, (c) Component of control panel and, (d) Control panel and sensor stand.

To evaluate the developed system, has set up one of the monitoring point of Pagla Sewage Treatment Plant (PSTP) of Dhaka WASA. The selected monitoring point is before of chlorination. After this point chlorination has done and water has discharged to environment. PSTP located in Dhaka, Bangladesh, is an essential facility for managing the city's domestic sewage (see Fig. 5). Originally built in 1968 with four facultative lagoons, PSTP underwent significant renovations and expansions in 1977 and again in 1992 with funding from Japan International Cooperation Agency (JICA). This expansion increased its capacity to handle up to 120,000 cubic meters of sewage per day, serving about 500,000 residents of Dhaka. The plant is located 8 km away from south-east portion of Dhaka city and about 1 km north from Buriganga River. The global positioning system (GPS) coordinate of the PSTP is 23.679379691452503 °N latitude, 90.454768190475°E longitude. As well as the GPS coordinate of BCSIR is 23.74006938328495 °N latitude, 90.3850450354044 °E longitude, where the testing has done with respect to standard sample (refer to Supplementary Fig. S1).

The treatment process at PSTP includes several stages: a grit chamber, primary sedimentation tanks, facultative lagoons, a chlorination system, and sludge lagoons. The power consumption by the system is very negligible amount of 29 W. The total cost for the proposed system was BDT 292000/- equivalent to USD 2445 including government vat and tax as well custom duty. In case of maintenance mainly the sensor probe cleaning. In every seven days we have cleaned the sensor probe with de-ionized water as well as the calibration of the sensor probe. The sensor probe was calibrated by following the in-house method for getting more accurate results. We have focused for the optimum cleaning frequency on various factors are manufacturer's recommendations, characteristics of the sewage water, visual inspections of the sensor probe and historical data analysis.

## 3 Result and discussion

In this study for monitoring and control the parameters of waters from cloud server total four screen has configured.

**Fig. 6:**
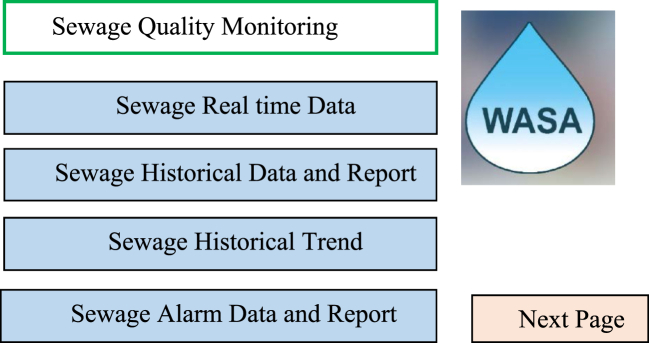
Introductory screen of cloud SCADA.

**Fig. 7:**
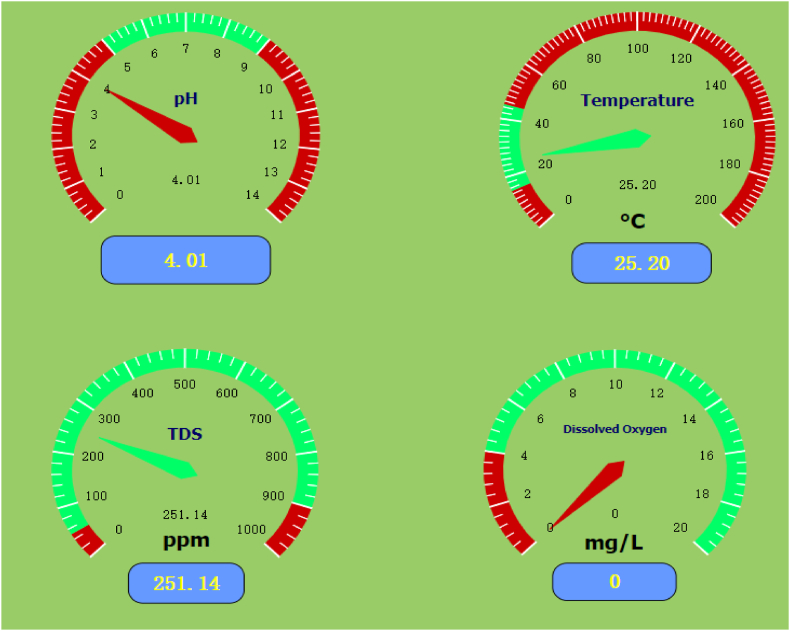
Cloud screen for real time data.

Within the four screens first screen is the introductory screen as shown in Fig. 6 where function switch has configured in such a way to move on to the next screen or directly to the designated screen. In Fig. 7 shows the typical real time screen for the standard sample. System performance, accuracy, and precision of water quality parameters were validated according to the testing standards: pH 4.0, TDS 250 ppm, DO 0 ppm, and water temperature 25 °C.

The historical data screen has configured in such a way to record the data in every 1 min.

Table 5 shows the typical historical data screen. Data can be possible to download in excel format by putting the time frame. The data accuracy of the output for TDS, water temperature, and DO is 0.1, while for pH, it is 0.2.

**Table 5:** Concentration of standard parameters at the designed system.

Data	TDS (mg/L)	Temperature ^0^C	DO (mg/L)	pH
2023-01-23 09:56:08	250.76	25.5	0.00	4.00
2023-01-23 09:57:08	249.55	25.6	0.00	4.00
2023-01-23 09:58:08	249.43	25.8	0.00	3.99
2023-01-23 09:59:08	250.53	24.7	0.01	4.01
2023-01-23 10:00:08	249.22	25.0	0.00	4.01
2023-01-23 10:01:08	249.94	24.6	0.00	4.01
2023-01-23 10:02:08	249.99	25.7	0.00	4.00
2023-01-23 10:03:08	249.60	25.3	0.00	3.98
2023-01-23 10:04:08	249.40	25.1	0.01	4.01
2023-01-23 10:05:08	249.34	24.9	0.00	3.99
2023-01-23 10:06:08	249.52	25.3	0.00	3.98
2023-01-23 10:07:08	251.25	25.0	0.00	4.01
2023-01-23 10:08:08	250.18	24.7	0.00	4.01
2023-01-23 10:09:08	250.98	24.7	0.00	4.00
2023-01-23 10:10:08	251.55	24.7	0.00	4.00
2023-01-23 10:11:08	250.82	25.1	0.00	4.00
2023-01-23 10:12:08	250.47	24.8	0.00	4.01

This result reveal that the sensors and IoT based monitoring system for water treatment plant (WTPs) is accurate and user frank. Table 6 shows the typical alarm list on the basis of standard sample.

**Table 6:**
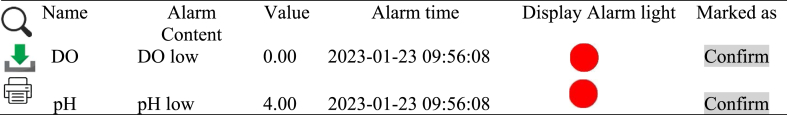
Shows the typical alarm list on the basis of standard.

In Supplementary Fig. S2 shows the historical trend for 48 h based on the standard sample value of Table 4. The trend has plotted on the basis of time which has shown on the X-axis, whereas on the left hand side Y-axis it shows the Temp (^0^C) and on the other hand DO (mg/L) and pH has shown on the right had side of the Y-axis.

### 3.1 Performance check of the designed IoT device

When confirming that the design device provides standard values for water quality parameters such as TDS (500 ppm), pH (4), and DO (4 ppm) accurately within a 0.1 to 0.2 variance, live sample values were recorded.

**Fig. 8:**
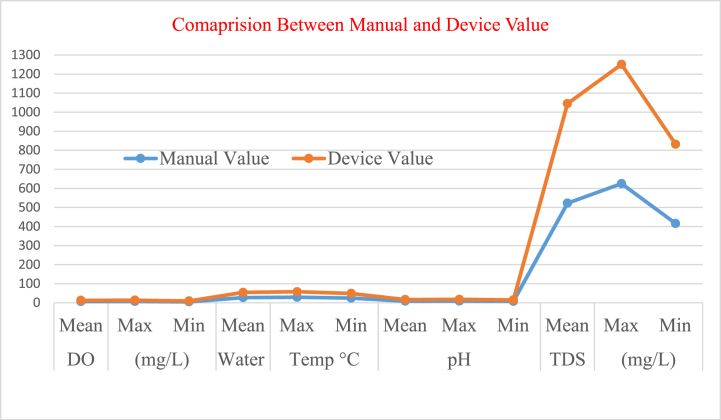
Comparison on between the manual and device value.

For additional confidence and verification, the manual water quality parameters values and device values set in WASA were compared. These comparison values were monitored over the year 2023, as shown in Supplementary Tables S2 and S3, and are also illustrated in Fig. 8. The descriptive statistics, including mean, maximum, and minimum values, were recorded month-wise from January to December. The highest concentrations were found in the dry season compared to the wet season due to the temperature sensitivity of these water quality parameters. During dry and cool conditions, the designed device accurately determined these water quality parameters.

According to Supplementary Tables S2–S4, and Fig. 8, the device determines water quality accurately, smartly, and easily both at the working station and in the remote control office.

## 4 Conclusion

The integration of IoT technology into water treatment plants represents a significant advancement in real-time water quality monitoring. The developed system, which utilizes PLC-based control and a robust sensor network, allows for continuous and precise monitoring of key water parameters. With the capability to send real-time data to a cloud server, the system ensures timely detection of any deviations from standard water quality, enabling prompt corrective actions. The minimal power consumption and cost-effectiveness of the system make it a viable solution for widespread deployment in water treatment facilities. The practical implications of IoT-based water quality monitoring are significant, especially in industries, municipalities, and environmental management. This system is remarkable for remote access, continuous data collection, immediate detection of issues, as well as high frequency data. The system also reduce the operational and labour costs.

This study demonstrates that the IoT-based monitoring system not only enhances the operational efficiency of water treatment plants but also contributes to better resource management and environmental sustainability. By providing accurate data and enabling remote monitoring, this system significantly reduces the need for manual intervention, thus minimizing operational costs and improving overall system reliability. Future research should focus on addressing the challenges related to sensor placement, data security, and system scalability to ensure broader adoption. The ongoing development and implementation of such technologies will undoubtedly play a crucial role in safeguarding public health by ensuring the continuous supply of safe drinking water.

## CRediT authorship contribution statement

**H.M. Forhad:** Writing – original draft, Visualization, Software, Methodology, Investigation, Formal analysis, Conceptualization. **Md. Ripaj Uddin:** Writing – review & editing, Writing – original draft, Project administration, Formal analysis, Data curation, Conceptualization. **R.S. Chakrovorty:** Investigation, Formal analysis. **A.M. Ruhul:** Formal analysis, Data curation. **H.M. Faruk:** Writing – review & editing, Methodology, Investigation, Formal analysis. **Sarker Kamruzzaman:** Writing – review & editing, Data curation. **Nahid Sharmin:** Writing – review & editing, Resources. **AHM Shofiul Islam Molla Jamal:** Methodology, Investigation. **Md. Mezba-Ul Haque:** Methodology, Investigation, Data curation. **AKM M Morshed:** Writing – review & editing, Validation, Supervision, Project administration.

## Data availability

All data are available in the manuscript.

## Declaration of competing interest

The authors declare that they have no known competing financial interests or personal relationships that could have appeared to influence the work reported in this paper.
