# Supplementary material for: IoT based real-time water quality monitoring system in water treatment plants (WTPs)
Source: Heliyon. 2024 Nov 29;10(23):e40746. doi: 10.1016/j.heliyon.2024.e40746 (PMC11652906; doi:10.1016/j.heliyon.2024.e40746)
Supplement: Multimedia component 1 [file mmc1.docx]

Table S1: Description of Cloud SCADA screen

| Page Name | Description of the page |
| --- | --- |
| Home Screen | Introduction and function switch to shift other screens. |
| Real time Screen | To monitor real time parameters. |
| Screen for historical data | This screen has used for monitoring historical data and to download the historical data in excel format. |
| Alarm Screen | According to configuration alarm will display for abnormal condition, historical alarm will be also displayed. For current a buzzer sound will ring and by clicking we have to confirm the alarm. |
| Real time trend screen | According to configuration real time trend will display by this screen. |
| Historical trend screen | Through this screen historical trend will be displayed according to configuration. |

Table S2: Titrimetric determine the concentration of water quality parameters month-wise over the year 2023

| Para |  | January | February | March | April | May | June | July | August | September | October | November | December | Mean |
| --- | --- | --- | --- | --- | --- | --- | --- | --- | --- | --- | --- | --- | --- | --- |
| DO  (mg/L) | Mean | 5.01 | 5.1 | 5.21 | 5.25 | 5.93 | 6.4 | 9.53 | 7.08 | 6.74 | 6.78 | 5.6 | 5.91 | 6.21 |
|  | Max | 4.51 | 4.5 | 4.2 | 4 | 8 | 8.1 | 8.91 | 8.8 | 7.92 | 7.6 | 6.5 | 6.1 | 6.60 |
|  | Min | 3.91 | 3.95 | 3.93 | 3.97 | 4 | 4.65 | 3.7 | 6.13 | 4.53 | 6.2 | 5.21 | 5.31 | 4.62 |
| Water  Temp °C | Mean | 23.62 | 22.23 | 28.25 | 28.23 | 28.72 | 28.52 | 29.83 | 29 | 29.35 | 28.65 | 25.23 | 23.23 | 27.07 |
|  | Max | 21.34 | 26.72 | 30.31 | 30.51 | 30.52 | 29.8 | 32.71 | 31.32 | 30.81 | 30.82 | 26.32 | 24.61 | 28.82 |
|  | Min | 19.5 | 19.6 | 25.7 | 26.2 | 26.4 | 27.3 | 27.4 | 27.31 | 25.7 | 25.21 | 21.4 | 21.2 | 24.41 |
| pH | Mean | 8.7 | 8.12 | 8.06 | 8.14 | 8.31 | 8.24 | 8.5 | 8.62 | 8.55 | 8.53 | 7.8 | 7.53 | 8.26 |
|  | Max | 8.68 | 8.65 | 9 | 8.9 | 8.91 | 8.92 | 9.3 | 9.6 | 9.86 | 8.95 | 7.8 | 7.62 | 8.85 |
|  | Min | 7.4 | 7.55 | 7.26 | 7.56 | 7.51 | 7.55 | 8.12 | 6.78 | 8.12 | 8.18 | 6.21 | 6.43 | 7.39 |
| TDS  (mg/L) | Mean | 525.4 | 606.2 | 762 | 793.2 | 599.5 | 585.2 | 485.7 | 423.5 | 393.5 | 363.6 | 345.5 | 384.9 | 522.35 |
|  | Max | 600 | 691.1 | 893 | 960 | 786.1 | 658 | 715.1 | 484 | 455 | 440.1 | 412 | 410 | 625.37 |
|  | Min | 467 | 544.1 | 689 | 700 | 30.2 | 483 | 398.1 | 380 | 363 | 300 | 311 | 324 | 415.78 |

Table S3: Digitally determine the concentration of water quality parameters month-wise over the year 2023

| Parameters | | January | February | March | April | May | June | July | August | September | October | November | December | Mean |
| --- | --- | --- | --- | --- | --- | --- | --- | --- | --- | --- | --- | --- | --- | --- |
| DO  (mg/L) | Mean | 5 | 5.12 | 5.2 | 5.27 | 5.95 | 6.41 | 9.55 | 7.09 | 6.76 | 6.79 | 5.62 | 5.9 | 6.22 |
|  | Max | 4.5 | 4.6 | 4.3 | 4.2 | 8.02 | 8.11 | 8.9 | 8.81 | 7.95 | 7.63 | 6.54 | 6.12 | 6.64 |
|  | Min | 3.9 | 3.98 | 3.95 | 3.99 | 4.01 | 4.66 | 3.72 | 6.15 | 4.55 | 6.22 | 5.2 | 5.3 | 4.64 |
| Water  Temp °C | Mean | 23.6 | 22.25 | 28.27 | 28.22 | 28.75 | 28.54 | 29.86 | 29.01 | 29.37 | 28.67 | 25.22 | 23.25 | 27.08 |
|  | Max | 21.39 | 26.7 | 30.3 | 30.5 | 30.5 | 29.9 | 32.7 | 31.3 | 30.8 | 30.8 | 26.3 | 24.6 | 28.82 |
|  | Min | 19.4 | 19.5 | 25.6 | 26 | 26.6 | 27.2 | 27.3 | 27.2 | 25.9 | 25.2 | 21.2 | 21.1 | 24.35 |
| pH | Mean | 8.29 | 8.13 | 8.05 | 8.13 | 8.3 | 8.26 | 8.52 | 8.64 | 8.54 | 8.52 | 7.79 | 7.52 | 8.22 |
|  | Max | 8.65 | 8.64 | 9.01 | 8.89 | 8.9 | 8.92 | 9.33 | 9.63 | 9.85 | 8.94 | 7.7 | 7.64 | 8.84 |
|  | Min | 7.39 | 7.54 | 7.25 | 7.55 | 7.5 | 7.58 | 8.12 | 6.78 | 8.12 | 8.18 | 6.2 | 6.45 | 7.39 |
| TDS  (mg/L) | Mean | 525.6 | 606.5 | 762.4 | 793.3 | 599.8 | 585.3 | 485.9 | 423.4 | 393.6 | 363.7 | 345.4 | 384.8 | 522.48 |
|  | Max | 601 | 691 | 892 | 959 | 786 | 657 | 715 | 483 | 456 | 440 | 410 | 412 | 625.17 |
|  | Min | 468 | 544 | 688 | 699 | 30.1 | 484 | 398 | 382 | 362 | 301 | 310 | 325 | 415.93 |

Table S4: Comparison concentration between manual and digital value

| Parameters | Statistic | Manual Value | Device Value |
| --- | --- | --- | --- |
| DO  (mg/L) | Mean | 6.22 | 6.21 |
|  | Max | 6.64 | 6.60 |
|  | Min | 4.64 | 4.62 |
| Water  Temp °C | Mean | 27.08 | 27.07 |
|  | Max | 28.82 | 28.82 |
|  | Min | 24.35 | 24.41 |
| pH | Mean | 8.22 | 8.26 |
|  | Max | 8.84 | 8.85 |
|  | Min | 7.39 | 7.39 |
| TDS  (mg/L) | Mean | 522.48 | 522.35 |
|  | Max | 625.17 | 625.37 |
|  | Min | 415.93 | 415.78 |


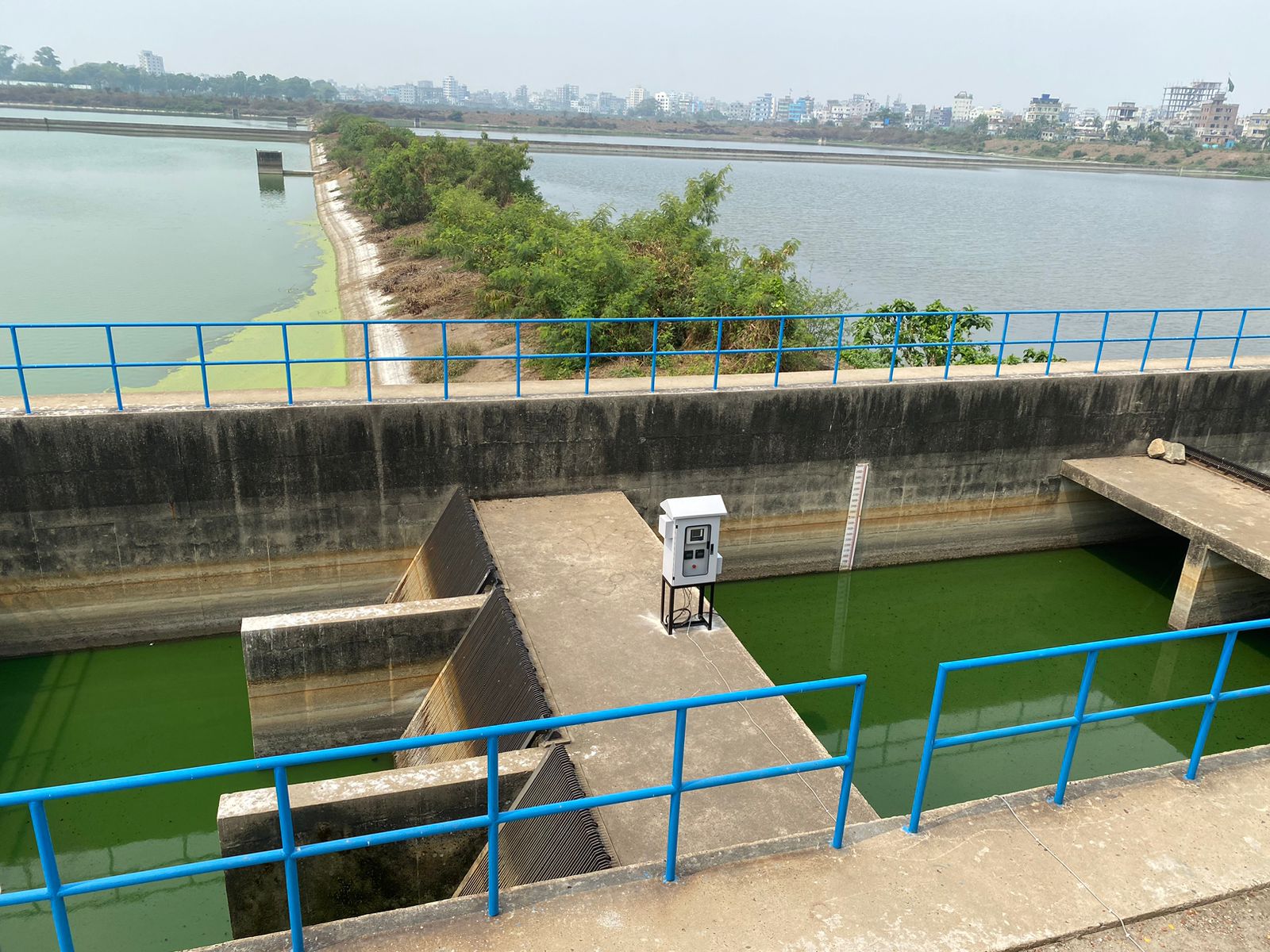


Fig. S1. Location of the control panel


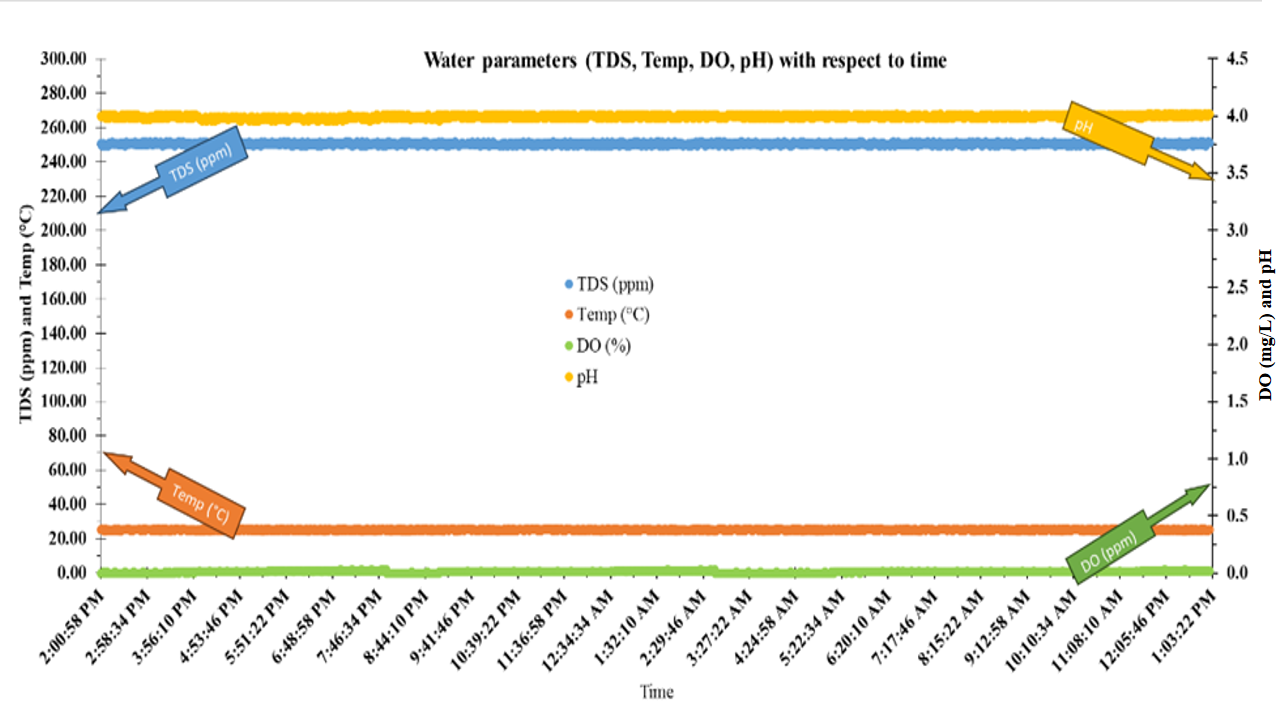


Fig. S2. Historical trend for the standard parameter
